# Possible SARS-coronavirus 2 inhibitor revealed by simulated molecular docking to viral main protease and host toll-like receptor

**DOI:** 10.2217/fvl-2020-0099

**Published:** 2020-06-12

**Authors:** Xiaopeng Hu, Xin Cai, Xun Song, Chenyang Li, Jia Zhao, Wenli Luo, Qian Zhang, Ivo Otte Ekumi, Zhendan He

**Affiliations:** 1 ^1^Department of Science and Education, Shenzhen Samii Medical Center, Shenzhen, China; 2 ^2^Guangdong Key Laboratory for Genome Stability & Human Disease Prevention, School of Pharmaceutical Sciences, Shenzhen Key Laboratory of Novel Natural Health Care Products, Innovation Platform for Natural Small Molecule Drugs, Engineering Laboratory of Shenzhen Natural Small Molecule Innovative Drugs, Shenzhen University Health Science Center, Shenzhen 518060, China

**Keywords:** COVID-19, GO and KEGG enrichment analysis, molecular docking, rutin, SARS-CoV-2 main protease, toll-like receptors, traditional antiviral medicinal plants

## Abstract

**Aim:** SARS-coronavirus 2 main protease (Mpro) and host toll-like receptors (TLRs) were targeted to screen potential inhibitors among traditional antiviral medicinal plants. **Materials & methods:** LeDock software was adopted to determine the binding energy between candidate molecules and selected protein pockets. Enrichment analyses were applied to illustrate potential pharmacology networks of active molecules. **Results:** The citrus flavonoid rutin was identified to fit snugly into the Mpro substrate-binding pocket and to present a strong interaction with TLRs TLR2, TLR6 and TLR7. One-carbon metabolic process and nitrogen metabolism ranked high as potential targets toward rutin. **Conclusion:** Rutin may influence viral functional protein assembly and host inflammatory suppression. Its affinity for Mpro and TLRs render rutin a potential novel therapeutic anti-coronavirus strategy.

SARS coronavirus 2 (SARS-CoV-2) first emerged in the city of Wuhan, China and progressively evolved into a severe pandemic [1]. The WHO proclaimed the outbreak of coronavirus disease-2019 (COVID-19) to be a Public Health Emergency of International Concern (PHEIC), which was the highest level of epidemic prevention in the world, suggesting its gravity [2]. Through international transportation, SARS-CoV-2 spread globally with more than four million reported cases and over 290,000 fatalities by 14 May 2020. The high morbidity and mortality rates were ascribed to the lack of effective drug treatment. COVID-19, for which SARS-CoV-2 is the etiological agent, poses a serious threat to human life during the continuation of the global outbreak.

Currently, the two main strategies for developing anti-CoV therapeutics have focused on virus-based or immunomodulatory treatments [3]. Numerous compounds directly targeting the virus inhibit the entry and/or replication of CoV *in vivo* or *in vitro*. For example, remdesivir and chloroquine target the RNA polymerase of CoV to exert a significantly strong inhibition [4]. Immunomodulators, such as either glucocorticoids to relieve symptoms of pulmonary inflammation by delaying the inflammatory cytokine storm, or interferon treatments to enhance the innate antiviral response, have been thought as excellent anti-CoV remedies [5,6].

In addition, numerous natural products have been suggested and tested for their antiviral effects. Augmentation of the interferon response by the administration of natural products has been reported [7–9]. In the past 20 years, a total of 109 natural constituents with antiviral or immunoregulation functions also have been reported and reviewed in [8]. Those 109 constituents were mainly isolated and purified from heat-clearing and detoxifying herbs and were classified as various kinds of alkaloids, terpenes, flavonoids or saponins. To screen potential SARS-CoV-2 inhibitors more effectively, the 109 constituents were selected as candidate molecules to dock with the crystal structure of SARS-CoV-2 main protease (Mpro) [10], which we tested herein via molecular docking software (Supplementary Table 1).

## Materials & methods

### Acquisition of chemical structure

The structures of 109 compounds [8] obtained from PubChem were saved as spatial data files, input into ChemBio3D Ultra 14.0 to minimize energy for the force field of the structure, and then saved in MOL2 molecular structure format.

### Docking method

The 3D structure of Mpro and a series of host toll-like receptors (TLRs) were obtained from the Research Collaboratory for Structural Bioinformatics protein data bank database. Protein data bank IDs of these molecules are as follows: Mpro (6lu7), TLR1 (6NIH), TLR2 (5d3i), TLR3 (1ziw), TLR4 (2z62), TLR5 (3v44), TLR6 (3a79), TLR7 (5gmf), TLR8 (4qc0) and TLR9 (3wpf). Inhibitor N3 was used as a ligand while analyzing the crystal structure of SARS-CoV-2 Mpro [10]. LeDock software was used to calculate the binding energy between ligands and targeted proteins because LeDock software presents significant reliability and accuracy compared with other docking software [11]. First, the input protein structure was provided with an added hydrogen for the sake of being charged electrically. Then, compound structures were input as ligands. Subsequently, the site of the grid box was identified according to the coordinates of the positive ligands in the target protein complex [10]. After the active pocket was well placed, LeDock calculations were performed for molecular docking. For each chemical structure, several docking poses were recommended through LeDock in addition to generate the binding energy. The optimum docking poses of each structure were applied for ranking, and the visualization of docking was performed with PyMOL 1.8 v4.4.0 (www.pymol.org) and LigPlot^+^ v.2.2 (www.ebi.ac.uk/thornton-srv/software/LigPlus/) software, respectively.

### Heatmap

The binding energy between 11 representative compounds and TLRs were visualized as a heatmap by MeV 4.9.0 based on the results presented in Table 2.

### Prediction for molecular mechanisms of rutin

The structure of rutin was loaded into Swiss Target Prediction (www.swisstargetprediction.ch/) to screen the potential target gene [12]. The functional annotation of the Database for Annotation, Visualization and Integrated Discovery (DAVID) v6.8 (https://david.ncifcrf.gov/) was applied for target gene annotation, and the Official Gene Symbol was chosen as the identifier in DAVID v6.8. Each target gene was analyzed via gene ontology (GO) and Kyoto Encyclopedia of Genes and Genomes (KEGG) [13]. The KEGG pathway enrichment bubble map was formed by R program v3.5.0.

## Results

### Docking between candidate molecular & Mpro

**Figure 1.**
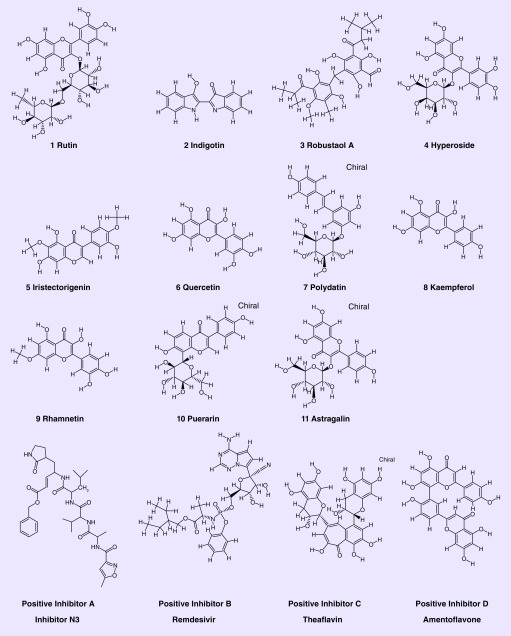
**The chemical structure of representative compounds.**

LeDock results depicted that flavonoid compounds (Table 1, Figure 1) displayed lower binding energy with Mpro compared with other structure types such as alkaloids, terpenes and saponins. Eleven compounds were identified with binding energies <-6.5 kcal/mol (Table 1), most of which were flavonoids. Of these, rutin demonstrated the lowest predicted binding energy in the active pocket of Mpro (-8.67 kcal/mol), even lower than the reported positive inhibitor (Table 1). Remdesivir was also regarded as the positive inhibitor toward Mpro with the lowest binding energy (-9.00 kcal/mol).

**Table 1.** **Docking results of representative compounds toward SARS-coronavirus 2 main protease (Mpro).**

Active Order	Compound	Molecular weight	Binding energy (kcal/mol)	Original plant	Ref.
1	Rutin	610	-8.67	*Forsythia suspense* (Thunb.) Vahl. *Houttuynia cordata* Thunb. *Prunella vulgaris* Linn. *Morus alba* L.	
2	Indigotin	262	-6.99	*Polygonum tinctorium* Ait. *Isatisin digotica* Fort.	
3	Robustaol A	474	-6.85	*Eucalyptus robusta* Smith.	
4	Hyperoside	464	-6.82	*Prunella vulgaris* Linn.	
5	Iristectorigenin	330	-6.8	*Belamcanda chinensis* (L.) DC.	
6	quercetin	302	-6.78	*Houttuynia cordata* Thunb. *Astragalus membranaceus* (Fisch.) *Pyrrosia lingua*(Thunb.)Farw. *Polygonum porfoliatum* L. *Patrinia villosa* (Thunb.) *Lonicera japonica* Thunb.	
7	Polydatin	390	-6.74	*Polygonum cuspidatum* Sieb. Et Zucc.	
8	Kaempferol	286	-6.68	*Polygonum tinctorium* Ait.	
9	Rhamnetin	316	-6.65	*Coptis chinensis* Franch.	
10	Puerarin	416	-6.63	*Pueraria lobata* Ohwi	
11	Astragalin	448	-6.51	Glycyrrhiza uralensis Fisch.	
Positve Inhibitor A	Inhibitor N3	680	-7.05	—	[10]
Positve Inhibitor B	Remdesivir	603	-9.00	—	[14]
Positve Inhibitor C	Theaflavin	564	-6.21	—	[15]
Positve Inhibitor D	Amentoflavone	538	-6.06	*Forsythia suspensa* (Thunb.) Vahl.	[16]

The affinity between flavonoids and targeted protein was much stronger compared with other types of compounds. This may be because the abundant phenolic hydroxyl group in flavonoids, especially the hydroxyl group in the sugar group of flavonoids, bind more easily with the heteroatoms of amino acids from Mpro (Figure 3). Rutin forms multiple hydrogen bonds with the main chain of residues like Phe-140, Glu-166, Thr-26, Leu-141, Ser-144, Cys-145 and His-163. In particular, Asn-142 and Gln-189 were thought to contribute to the hydrophobic interactions with rutin (Figure 3).

**Figure 2.**
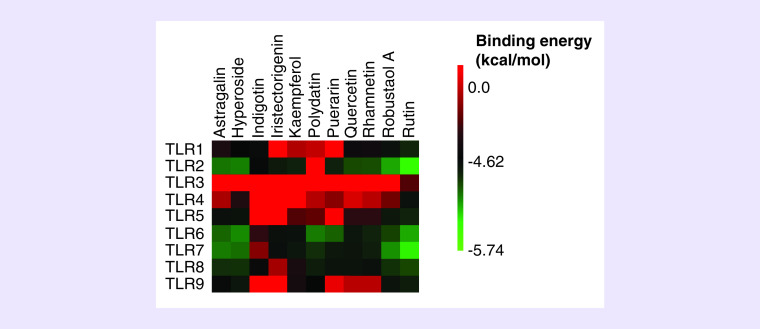
**The hot map of docking between representative compounds and toll-like receptors.** The greener square represents lower binding energy between TLR and compounds, indicating the potential interactions. In contrast, the red square means the interactions between molecules and targets are extremely impossible. TLR: Toll-like receptor.

**Figure 3.**
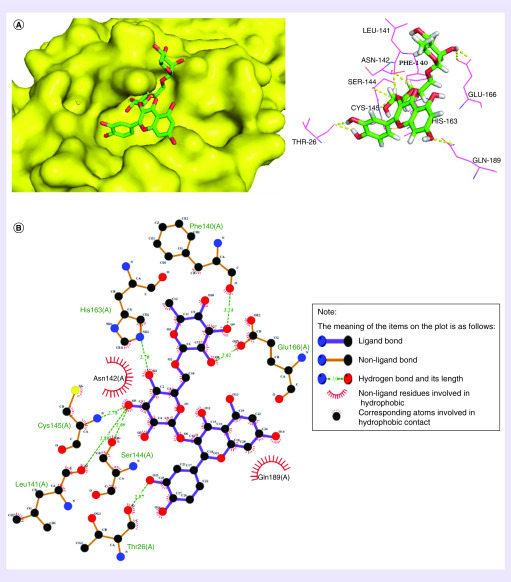
**The docking model between rutin and SARS-coronavirus 2 main protease (Mpro) is exhibited as 3D interaction diagram through the LeDock server.** The yellow dash lines represented potential interactions between the amino acid residues of Mpro and rutin. The name of binding amino acid residues are labeled with abbreviations.

### Docking between 11 selected compounds & TLRs

TLRs play an important role in mediating the inflammatory response and host-based anti-CoV activity. The pocket site of TLR2, TLR6 and TLR7 presented potential combinations between rutin with binding energies of <-8 kcal/mol (Table 2, Figure 2). TLRs generally stimulate pro-inflammatory and antiviral host pathways. These potential bindings indicate two possible activities: antagonistic or stimulatory. For patients with COVID-19, this may provide a dual benefit, both preventing over-inflammation and restoring innate antiviral immunity [3].

**Table 2.** **Docking result of representative compounds toward toll-like receptors.**

Binding energy (kcal/mol)	Astragalin	Hyperoside	Indigotin	Iristectorigenin	Kaempferol	Polydatin	Puerarin	Quercetin	Rhamnetin	Robustaol A	Rutin
TLR1	-5.51	-5.74	-5.89	-4.55	-4.95	-4.89	-4.51	-5.65	-5.63	-6.11	-6.79
TLR2	-7.99	-8.12	-5.83	-6.45	-6.65	0	-6.65	-7.42	-7.43	-8.63	-9.76
TLR3	-4.26	-4.4	-3.37	-3.9	-3.54	-4.62	-3.84	-4.34	-4.43	-4.48	-5.29
TLR4	-4.97	-5.49	-3.6	-4.43	-4.1	-4.94	-5.09	-4.82	-4.92	-5.17	-6.1
TLR5	-6.07	-6.13	-4.58	-4.58	-5.29	-5.23	-4.67	-5.42	-5.43	-6.34	-6.72
TLR6	-7.79	-8.28	-5.41	-6.04	-6.11	-8.1	-7.71	-6.29	-6.7	-7.28	-8.66
TLR7	-8.09	-7.88	-5.1	-5.95	-6.29	-6.94	-6.37	-6.24	-6.55	-8.33	-9.58
TLR8	-6.93	-6.96	-5.68	-4.99	-5.53	-6.51	-6.18	-6.2	-6.02	-7	-7.31
TLR9	-5.84	-6.26	-3.94	-4.37	-5.57	-5.74	-4.76	-4.93	-4.93	-6.21	-6.51

TLR: Toll-like receptor.

### GO & KEGG enrichment analysis of potential targets toward rutin

**Figure 4.**
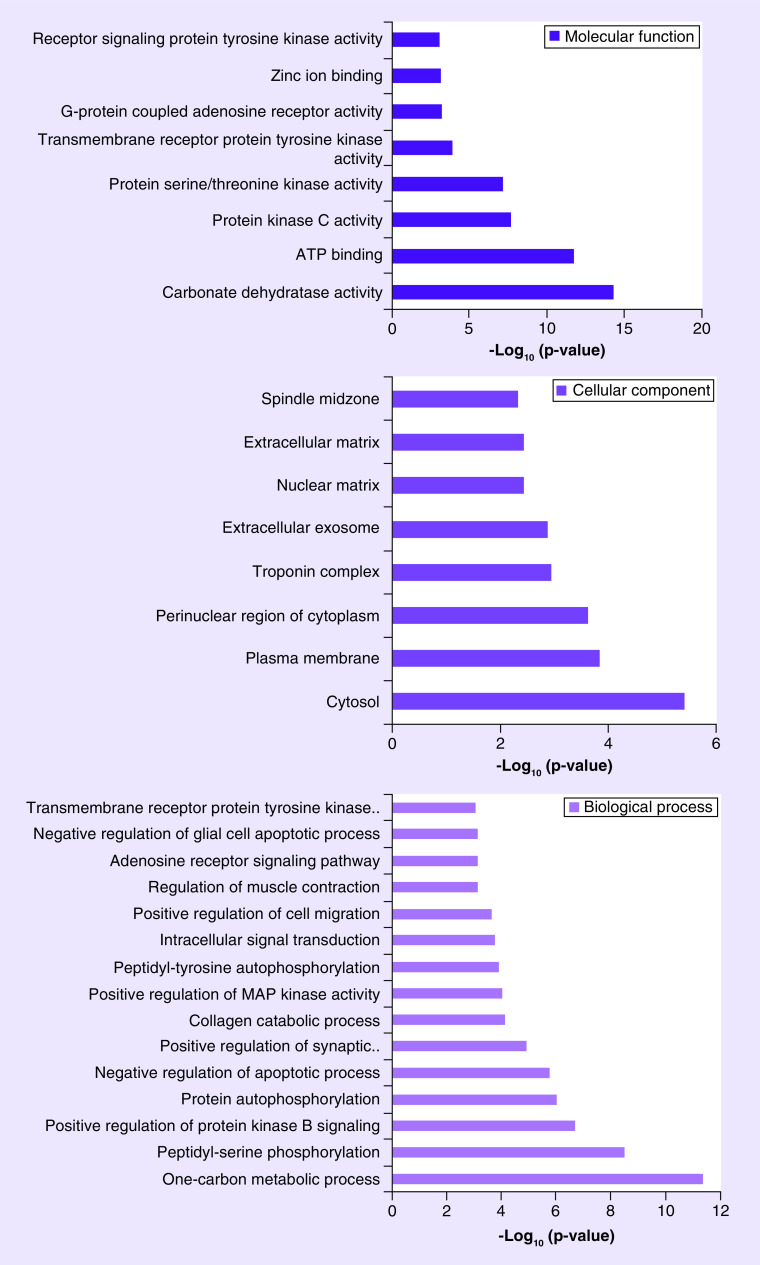
**Gene ontology enrichment analysis of the targets toward rutin.** In term of molecular function, the predicted targets mainly participate into the carbonate dehydratase activity. As for cellular component, the predicted targets mainly occurred in cytosol. During biological process, one-carbon metabolic is thought to be the major process.

The Swiss Target Prediction yielded more than 100 target genes for rutin. GO annotation output was classified into three enrichment branches: biological process (BP), cellular component and molecular function (Figure 4). Carbonate dehydratase and protein kinase C activity were of greater significance in rutin mediating BP. As for cellular component, the rutin-predicted target mainly participated in the cytosol and troponin complex. The one-carbon metabolic process and peptidyl-serine phosphorylation were thought to be closer interrelated with rutin-predicted targets during molecular function.

**Figure 5.**
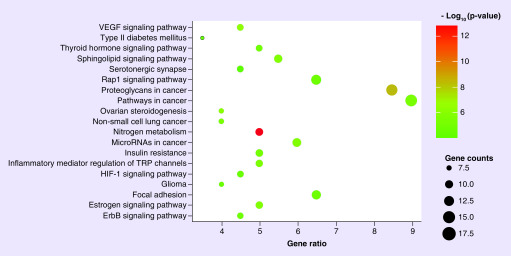
**Analysis of Kyoto Encyclopedia of Genes and Genome enrichment in related pathways as targets of rutin.** The diameter of the circle represented the accounts of rutin target gene. The deeper shadow of orange represents the greater difference in significance. Rutin-related target genes (CA14, CA9, CA13, CA7, CA12, CA6, CA4, CA3, CA2 and CA1) were assigned to nitrogen metabolism signaling pathway with significant differences.

The KEGG pathway showed potential rutin targets in pathways such as nitrogen metabolism, proteoglycans in cancer, Rap1 signaling and VEGF signaling (Figure 5). These pathways are closely related with lung inflammation, suggesting that the application of rutin may exert suppression of inflammation during CoV infection [17].

## Discussion

Virally induced pneumonia has been associated with the secretion of pro-inflammatory cytokines. Cytokine storms are thought to be the main cause of progressive respiratory failure via induction of inflammatory cell infiltration and alveolar damage [18]. Pro-inflammatory cytokines IL-1 and IL-6 are believed to play catalytic roles in viral inflammation [19]. Recent studies have shown the potential of therapeutic anti-inflammatory cytokines, including IL-37 or IL-38, to demonstrate immunosuppressive activity and alleviate lung inflammation, fever and fibrosis [20], suggesting the possibility that viral inflammation may be inhibited by anti-inflammatory cytokines. Cytokine signaling is highly associated with the activation of TLRs [21]. A series of studies reveal that blocking TLR signaling also prevents cytokine storms, indicating a potential therapeutic target for SARS-CoV-2-induced inflammation. Interestingly, men seem to be more vulnerable than women to SARS-CoV-2 infection due to the differences in immune responses to innate immunity. Triggering TLR7 to produce interferon appears to occur more readily in women than in men [22].

The antiviral properties of natural compounds via regulation of the innate antiviral response provides a promising therapy for the clinical treatment of SARS-CoV-2 infection. Mpro, a coronavirus main protease, is a critical enzyme mediating the production of CoV functional proteins [23,24]. Recently, a high-resolution crystal structure of Mpro was identified, making it an attractive target for drug discovery [10]. This enabled us to use LeDock to determine the binding capacity between SARS-CoV-2 Mpro and the 109 compounds previously identified in natural products.

The potential immunomodulatory effects of 11 of these compounds were determined via docking with TLR1 through TLR9 (Table 2). TLRs are pattern recognition receptors that recognize pathogen-associated molecular patterns [21]. When TLRs recognize an exogenous ligand, the innate immune response is activated and begins activation of the adaptive response, causing antiviral immunity or even excessive inflammatory response. In this study, we discovered that rutin not only binds tightly to Mpro, but also acts as a regulator of TLR2, TLR6 and TLR7 (Figure 2).

In terms of the source of rutin, traditional Chinese medicines such as *Forsythia suspense*, *Houttuynia cordata*, *Prunella vulgaris* or *Morus alba*, possess rutin as an active constituent (Table 1). Apart from traditional Chinese medicines, tea leaves and apples also contain ample rutin [25]. *Fagopyrum* species such as buckwheat are the richest source of the flavonoid rutin [26]. Rutin, also known as vitamin P, has been widely used as an antioxidant in the food processing industry. Therefore, it would be easy to ingest rutin in daily meals. In addition, many supplementary complex vitamins contain rutin [27]. Therefore, it would be beneficial for our body to ingest complex vitamins, especially those containing rutin, during the outbreak of COVID-19.

The possible involvement of cellular BPs and pathways of rutin were briefly discussed based on bioinformatics analysis. We found that the one-carbon metabolic process ranked high as a potential target of rutin (Figure 4). A previous study showed that carbohydrates may serve as receptor determinants when SARS-CoV-2 attaches to host cells [28]. Therefore, the effect of rutin-related one-carbon metabolic processes deserves further research during viral infection. The functional annotation of GO and the enrichment analysis indicated that related pathways (nitrogen metabolism, proteoglycans in cancer, Rap1 signaling pathway, VEGF signaling pathway) may play critical roles in the anti-inflammatory response with rutin (Figure 5). Interestingly, nitric oxide (NO) had been reported to inhibit the SARS-CoV-2 viral RNA production [29]. Besides this, NO or its derivatives may also influence palmitoylation of the nascently expressed viral spike (S) protein, blocking the reorganization process of angiotensin converting enzyme 2. Thus, anti-CoV medicine may be developed by targeting NO-related enzyme proteins. However, the role of rutin in mediating nitrogen metabolism during SARS-CoV-2 infection requires further studies.

All of the data provided in this paper are based on pure bioinformatic analyses. Therefore, the results should not be applied clinically without further evaluation of the potential inhibitors via experimental confirmation *in vitro* and *in vivo*.

These docking results must be validated by a process first involving expression and purification of the SARS-CoV-2 main protease via recombinant gene expression *in vitro*. Then a tryptophan-based fluorescence method reported recently could be used to confirm the interaction between Mpro and rutin, or other potential inhibitors [30]. This process would be direct and simple and available in biosafety level 1 laboratories. Furthermore, biochemical and cell-based assays must be applied to evaluate the solubility, toxicity and pharmacodynamic properties of rutin toward SARS-CoV-2 Mpro. If the EC_50_/IC_50_ are high at indicated dosages, the antiviral activity of rutin or other potential inhibitors would be further studied *in vivo*, especially in animal models that express human angiotensin converting enzyme (ACE2), the putative cellular receptor for SARS-CoV-2 [31]. To clarify the anti-inflammation mechanism of rutin toward SARS-CoV-2, knockout mice with deficiencies in T cells, B cells and/or natural killer (NK) cells could also be utilized [3]. To date, however, evaluation of anti-CoV activity is only available in biosafety level 3 laboratories, where experiments are highly technically demanding.

## Conclusion

Flavonoid compounds, particularly rutin, exhibited good characteristic of binding with SARS-CoV-2 Mpro and TLRs, indicating it as a novel therapeutic option via virus-based and host-based anti-CoV strategies.

**Summary points**

- Eleven compounds (Table 1) with lower binding energy were identified as SARS-coronavirus 2 potential inhibitors.
- Rutin was highlighted not only because it fits snugly into the substrate-binding pocket of Mpro, but also because it presents a strong interaction with TLR2, TLR6 and TLR7.
- Gene ontology suggested that carbonate dehydratase and protein kinase C activity are of greater significance in rutin-mediating biological processes. The rutin-predicted target mainly participates in the troponin complex of the cellular component category. One-carbon metabolic process and peptidyl-serine phosphorylation are more closely interrelated with rutin in the molecular function category.
- Kyoto Encyclopedia of Genes and Genome pathway analysis showed that rutin exerts anti-inflammatory activity via nitrogen metabolism.
